# Increase in Oral Streptococcal Endocarditis Among Moderate-Risk Patients: Impact of Guideline Changes on Endocarditis Prevention

**DOI:** 10.1016/j.jacadv.2024.101266

**Published:** 2024-09-06

**Authors:** Jana Epprecht, Bruno Ledergerber, Michelle Frank, Matthias Greutmann, Mathias van Hemelrijck, Lilly Ilcheva, Maria Padrutt, Bernd Stadlinger, Mutlu Özcan, Thierry Carrel, Barbara Hasse

**Affiliations:** a Department of Infectious Disease and Hospital Epidemiology, University Hospital Zurich, Zurich, Switzerland; b Department of Cardiology, University Hospital Zurich, Zurich, Switzerland; c Department of Cardiac Surgery, University Hospital Zurich, Zurich, Switzerland; d Clinic of Cranio-Maxillofacial and Oral Surgery, University of Zurich, Zurich, Switzerland; e Clinic of Masticatory Disorders and Dental Biomaterials, Center for Dental Medicine, University of Zurich, Zurich, Switzerland; f Department of Cardiac Surgery, University Hospital Basel, Basel, Switzerland

**Keywords:** antibiotic prophylaxis, bicuspid aortic valve, invasive dental procedures, mitral valve prolapse, oral streptococci

## Abstract

**Background:**

The well-established connection between oral bacteria and infective endocarditis (IE) has prompted discussions about using antibiotic prophylaxis (AP) before invasive dental procedures. In 2007/2008, guidelines restricted AP from moderate and high-risk to exclusively high-risk patients.

**Objectives:**

The authors aimed to assess whether the proportion of oral streptococcal IE increased in moderate-risk patients using University Hospital Zurich data from 2000 to 2022.

**Methods:**

Adult IE patients were categorized into risk groups based on European Society of Cardiology and Swiss guidelines. The investigation focused on analyzing the proportion of oral streptococcal IE across different risk groups in two distinct periods (1: 2000-2008; 2: 2009-2022). Logistic regression models, adjusted for various factors, were employed.

**Results:**

Of 752 IE cases, 163 occurred in period 1, and 589 in period 2. Oral streptococci caused 22% of cases. Proportions of streptococcal IE in period 1 versus period 2 were 24% versus 16% in high-risk, 24% versus 39% in moderate-risk, 33% versus 7% in low-/unknown-risk, and 18% versus 14% in no-risk patients. Compared to the other risk groups, the moderate-risk group had a 22% higher chance of oral streptococcal IE in period 2. After multivariable adjustment, moderate-risk patients had twice the risk of oral streptococcal IE compared to period 1 (OR: 2.59 [95% CI: 1.16-5.81]). Among moderate-risk conditions, congenital valve anomalies were associated with oral streptococcal IE (unadjusted OR: 2.52 [95% CI: 1.71-3.71]).

**Conclusions:**

Oral streptococcal IEs increased in the moderate-risk group of patients after the AP guideline change. Exploring the potential necessity for expanding AP indications to certain patient groups with congenital valve anomalies may be warranted.

Infective endocarditis (IE) is an infection of the endocardium, typically affecting degenerated or congenitally malformed cardiac valves, prosthetic valves, or intracardiac devices. While IE is rare, associated mortality rates are high and can reach up to 30% in affected patients within the first year after diagnosis.^1^ In 1909, the British physician Thomas Horder identified the oral cavity as a primary entry point for bacteria. In 1948, initial studies demonstrated the efficacy of penicillin in IE prophylaxis.^2^^,^^3^ In 1955, the American Heart Association (AHA) first recommended antibiotic prophylaxis (AP) administration prior to invasive dental or medical procedures in at-risk patients, aiming to prevent bacterial adhesion to damaged endothelium during transient bacteremia.^4^

Given the evidence either supporting or discouraging the use of AP prior to dental procedures for IE prevention is very limited,^5^ AHA ^6^ and the European Society of Cardiology (ESC) ^7^ revised their guidelines in 2007/2009, restricting AP from moderate- and high-risk patients to only high-risk patients undergoing invasive dental procedures. Switzerland adopted this policy in 2008.^8^ With the doubling of IE incidence in Europe over the past 2 decades,^9^ investigating the impact of refraining from AP in at-risk individuals becomes crucial. Population-based studies examining changes in overall IE incidence and specifically streptococcal IE before and after the guideline update have yielded conflicting results.^10^, ^11^, ^12^, ^13^, ^14^, ^15^, ^16^, ^17^, ^18^ Consequently, no immediate action regarding AP management was considered necessary.^19^ While many studies focused on streptococcal IE in a general sense, only a few have explored the implications among different IE risk groups. Moreover, recent research suggests a need for re-evaluating risk stratification, as certain conditions currently classified as moderate risk exhibit a similar risk of developing and succumbing to IE as some high-risk conditions.^20^ This study aims to evaluate whether the proportion of streptococcal IE has increased among patients at moderate risk for IE since the discontinuation of AP in this patient group in 2008.

## Methods

### Study design, setting, and ethics approval

This single-center observational study was conducted at the University Hospital of Zurich in Switzerland (USZ), a tertiary referral center and teaching hospital with approximately 900 beds. We retrospectively included all adult individuals (≥18 years) referred to our center with IE from 2000 to 2017. The necessity of securing informed consent from retrospectively included patients was exempted by the ethics committee owing to the extended inclusion period and the observational character of the study. In instances where patients declined further use of data, they were excluded from the analyses. Starting from January 2018, we included patients from the ENdovascular and cardiac VALVE infection registry (ENVALVE), a prospective observational cohort study of adult patients with possible or confirmed IE. In ENVALVE, we monitor IE patients for a minimum of 1 year. All participants in the prospective cohort study provided their informed consent for the continued use of data. This study received approval from the Ethics Committee of the Canton of Zurich, Switzerland (KEK ZH 2014-0461; BASEC 2017-01140).

### Study participants

Patients aged 18 or older with any type of IE were included, covering the period from January 1, 2000, to September 30, 2022.

### Data collection

Study data were collected and managed using REDCap electronic data capture tools hosted at University Hospital Zurich.^21^^,^^22^ REDCap (Research Electronic Data Capture) is a secure, web-based software platform designed to support data capture for research studies, providing 1) an intuitive interface for validated data capture; 2) audit trails for tracking data manipulation and export procedures; 3) automated export procedures for seamless data downloads to common statistical packages; and 4) procedures for data integration and interoperability with external sources. Our data collection included demographic, clinical, laboratory, microbiology, imaging and treatment information utilizing the local hospital information system as a data source. Additionally, we captured risk factors for IE and 1-year outcomes including mortality, repeated cardiac surgery, relapse, and reinfection.

### Definitions and measures

Alcohol use was stratified based on the World Health Organization’s definition of severe, moderate, and light use. Obesity was defined as a body mass index exceeding 30 kg/m2.

The IE diagnosis was based on the version of the Duke criteria ^23^^,^^24^ valid at the time point of the assessment for suspected IE. To investigate the potential impact of the updated recommendations on AP issued in 2008,^8^ we divided the observation period into two time periods (period 1: January 1, 2000 through December 31, 2008; and period 2: January 1, 2009 through September 2022). Cases were allocated to these periods based on the date of IE diagnosis. As depicted in the Central Illustration, patients were categorized into high, moderate, and low/unknown risk for IE, according to the criteria outlined in the American, European, and Swiss guidelines.^6^, ^7^, ^8^^,^^25^, ^26^, ^27^, ^28^, ^29^ In case of multiple risk factors, we classified patients based on the highest applicable risk group for each instance. Risk factors present at the time of IE diagnosis were tallied irrespective of the patient's prior awareness of them. The moderate-risk group includes those individuals who discontinued AP for invasive dental procedures following the guideline update.

**Central Illustration:**
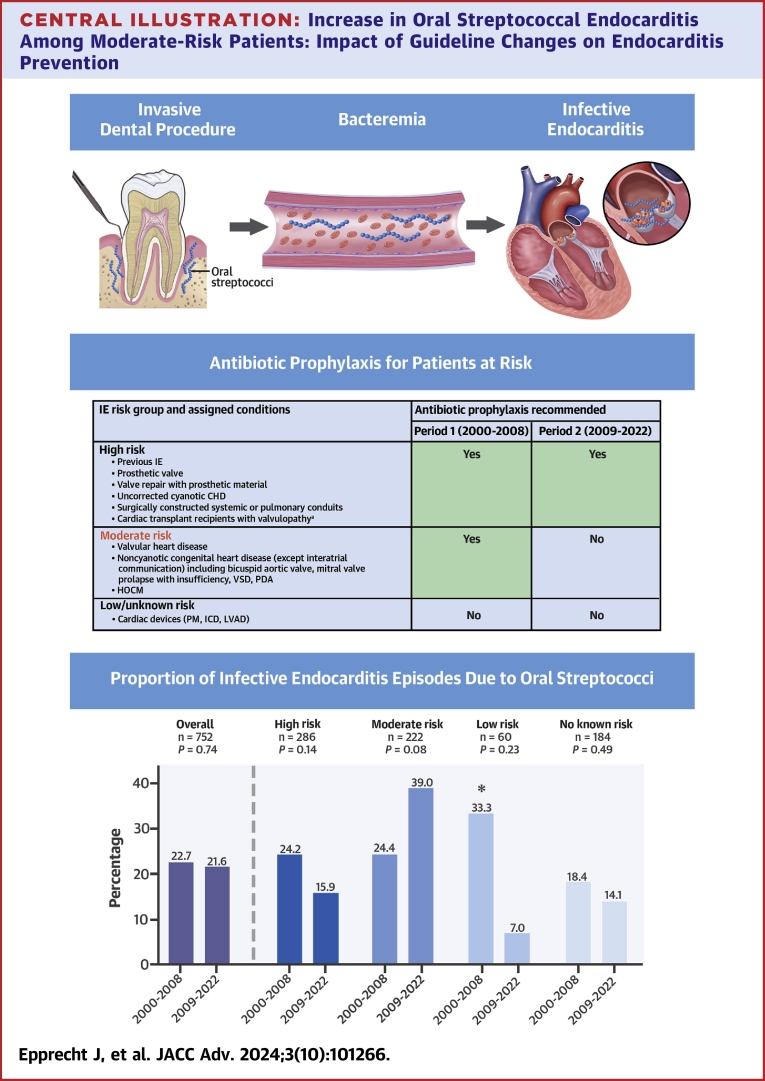
**Increase in Oral Streptococcal Endocarditis Among Moderate-Risk Patients: Impact of Guideline Changes on Endocarditis Prevention** Infective Endocarditis (IE) risk stratification and corresponding Antibiotic Prophylaxis (AP) Indications for invasive dental procedures before and after the guideline change are shown in the upper part of the illustration. Below, the proportion of IE episodes attributed to oral Streptococci is presented for the periods before and after the change in AP guidelines, both overall and stratified by IE risk groups. While we observed no change in the overall proportion, there was a notable increasing trend in the moderate-risk group and a corresponding decreasing trend in the high-, low-, and no-risk groups. ^a^AP recommended in the American and Swiss guidelines.^6^^,^^8^ However, the 2009 ESC guidelines ^7^ did not recommend AP for this group of patients. ∗Please note unequal distribution of patients in the low-risk group, period 1: 3 patients, period 2: 57 patients. CHD = congenital heart disease; ESC = European Society of Cardiology; HOCM = hypertrophic obstructive cardiomyopathy; ICD = implantable cardioverter defibrillator; LVAD = left ventricular assist device; PDA = persistent ductus arteriosus; PM = pacemaker; VSD = ventricular septal defect; other abbreviation as in Figure 1.

We designated manipulation of the gingival/periapical region of the teeth and perforation of the oral mucosa within 12 weeks preceding the diagnosis of IE as a high-risk dental procedure. Oral hygiene was documented according to treating physicians’ and/or dentists’ assessment. Poor oral hygiene was defined as inadequate practices in mouth, teeth, and gum care, including irregular or improper brushing, flossing, and dental check-ups, leading to the accumulation of plaque, bacteria, and substances contributing to issues like cavities, gum disease, and bad breath. Streptococci play a significant role in the composition of the oral microbiota. We categorized *Streptococcus mitis, S oralis, S parasanguinis, S sanguinis, S gordonii, S intermedius, S anginosus, S salivarius, S mutans* as oral streptococci. [23] *Granulicatella* spp, *Abiotrophia* spp, and *Gemella* spp, streptococcus-like organisms, were also included due to phenotypic and pathogenic similarity. Conversely, *S bovis, S equinus, S pyogenes, S agalactiae, S dysgalactiae, S equisimilis, S zooepidemicus, and S pneumoniae* were assigned to the group of non-oral streptococci.

### Statistical analyses

We compared episodes of IE between period 1 and period 2. Qualitative variables were presented as numbers and percentages, while quantitative variables were expressed as median (IQR). Categorical variables were compared using Fisher’s exact test, and continuous variables were assessed with nonparametric methods (Wilcoxon rank-sum test). We calculated the proportion of IE episodes due to oral streptococci and all streptococci across different risk conditions, utilizing the total number of IE episodes in patients with corresponding risk conditions in the data set as denominator. We analyzed proportion differences between the time periods among participants at moderate risk versus all other risk groups by calculating point estimates and 95% CIs of the difference between means. This grouping aimed to evaluate changes in the patient subset affected by the guideline change (ie, the moderate-risk group) with the other unaffected risk groups serving as a baseline. Univariable and multivariable logistic regression models were fitted for the odds of IE due to streptococci. Factors with a *P* < 0.60 were included in the multivariable models. The final models were adjusted for age (per 10 years older), sex, Charlson comorbidity index (CCI), intravenous drug use (IVDU), poor oral hygiene, high-risk dental procedures, IE risk category, and time period. Estimates between the outcome and variables are presented as ORs with 95% CIs. Standard errors for differences in means and logistic regression analyses were adjusted for patient clusters. We also calculated Kaplan-Meier estimates comparing survival in the two periods using the log-rank test. All analyses were performed using Stata 17.0 (Stata Corporation.

## Results

### Study population

**Figure 1:**
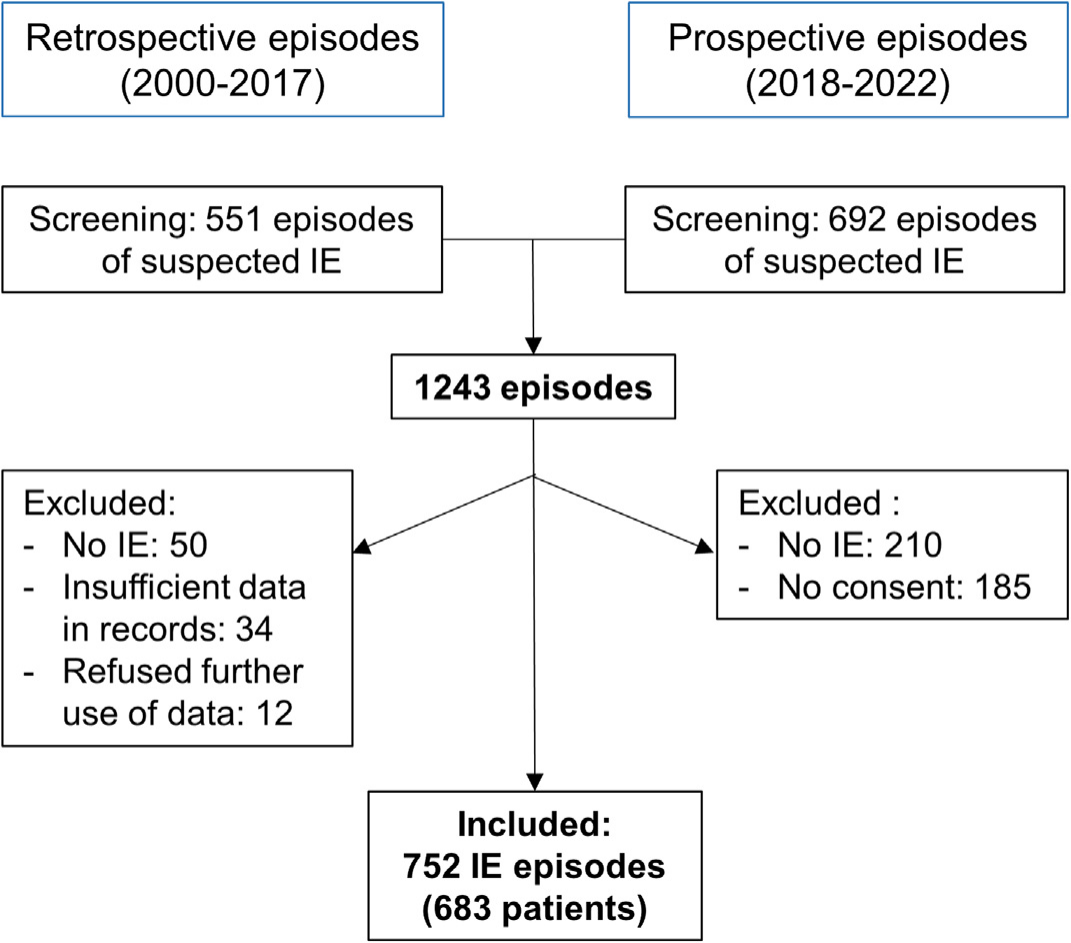
**Study Flowchart** Suspected episodes of infective endocarditis were retrospectively screened from 2000 to 2017 and prospectively from 2018 to 2022. The flowchart illustrates the reasons for exclusion. In total, 752 episodes were included in the analysis. IE = infective endocarditis.

We screened 1,243 suspected IE episodes from January 1, 2000, through September 30, 2022. Of these, 491 episodes were excluded, primarily due to the dismissal of IE diagnosis or the absence of patient consent (refer to Figure 1). The analysis ultimately included 752 IE episodes from 683 individual patients.

Table 1 displays patient characteristics stratified by period. The majority of patients were male constituting 75.8% of IE episodes, with a median age of 60 years (IQR: 44-71 years). In the second period, several chronic comorbidities were notably more prevalent, while IVDU was more common in the first period (n = 35 [21.5%] vs n = 64 [10.9%]). The moderate-risk category comprised 223 IE episodes (29.7%), primarily involving patients with acquired valvular dysfunction (n = 127, 16.7%) and congenital valve anomalies (n = 117, 15.6%). The latter group mainly consisted of patients with either a bicuspid aortic valve (BAV) (n = 59, 7.9%) or mitral valve prolapse (MVP) with insufficiency (n = 55, 7.3%). Overall, *Staphylococcus aureus* emerged as the most frequent causative pathogen, accounting for 260 episodes (34.6%), followed by streptococci (n = 230, 30.6%) and enterococci (n = 77, 10.2%). Surgical intervention for IE was conducted in 453 cases (60.2%), and the 1-year mortality rate was 22.5% (95% CI: 19.3%-26.1%).

**Table 1:** Baseline Characteristics of 752 Patients With Infective Endocarditis

	Total 2000-2022 (n = 752)	Period 1 2000-2008 (n = 163)	Period 2 2009-2022 (n = 589)	*P* Value
Male	570 (75.8)	128 (78.5)	442 (75.0)	0.409
Age, y	60 (44-71)	54 (37-66)	61 (47-72)	<0.001
BMI, median kg/m^2^	24.6 (21.9-27.8)	24.0 (20.9-26.9)	24.7 (22.2-28.1)	0.012
Smoking status				<0.001
Never	272 (36.2)	51 (31.3)	221 (37.5)	
Former	190 (25.3)	30 (18.4)	160 (27.2)	
Current	216 (28.7)	50 (30.7)	166 (28.2)	
Unknown	74 (9.8)	32 (19.6)	42 (7.1)	
Alcohol consumption				0.014
None/light	542 (72.1)	100 (61.4)	442 (75.0)	
Moderate/severe	80 (10.6)	23 (14.1)	57 (9.7)	
Unknown	130 (17.3)	40 (24.5)	90 (15.3)	
Comorbidities and risk factors for IE				
CCI	1 (0-3)	2 (0-4)	1 (0-3)	<0.001
Obesity	124 (16.5)	17 (10.4)	107 (18.2)	0.008
Atrial fibrillation	182 (24.2)	19 (11.7)	163 (27.7)	<0.001
Active cancer	98 (13.0)	10 (6.1)	88 (15.0)	0.002
Congestive heart failure	159 (21.1)	21 (12.9)	138 (23.4)	0.003
Myocardial infarction	79 (10.5)	12 (7.4)	67 (11.4)	0.151
COPD	46 (6.1)	5 (3.1)	41 (7.0)	0.067
Diabetes mellitus	123 (16.4)	17 (10.4)	106 (18.0)	0.023
Dialysis	22 (2.9)	2 (1.2)	20 (3.4)	0.192
Hypertension	384 (51.1)	63 (38.7)	321 (54.5)	<0.001
Immunosuppression	70 (9.3)	17 (10.4)	53 (9.0)	0.645
IVDU	99 (13.2)	35 (21.5)	64 (10.9)	0.001
Opioid substitution therapy	94 (12.5)	26 (16.0)	68 (11.5)	0.141
Liver cirrhosis	30 (4.0)	3 (1.8)	27 (4.6)	0.172
Other infectious disease	228 (30.3)	31 (19.0)	197 (33.5)	<0.001
Invasive procedures, oral health, and antibiotic prophylaxis				
Poor oral hygiene	118 (15.7)	34 (20.9)	84 (14.3)	0.051
Documented dental procedure before IE	59 (7.9)	12 (7.4)	47 (8.0)	0.871
Documented ORL, pneumological, urological, or gastrointestinal procedure before IE	48 (6.4)	9 (5.5)	39 (6.6)	0.719
Prophylaxis given	31 (4.1)	5 (3.1)	26 (4.4)	0.655
Type of prophylaxis				0.665
Amoxicillin ± clavulanate	16 (2.1)	2 (1.2)	14 (2.4)	
Other	15 (2.0)	3 (1.8)	12 (2.0)	
Endocarditis risk groups 6, 7, 8				
High-risk condition	286 (38.0)	66 (40.5)	220 (37.4)	0.467
Prior IE	118 (15.7)	39 (23.9)	79 (13.4)	0.002
Prosthetic valve replacement	188 (25.0)	40 (24.5)	148 (25.1)	0.919
Valve repair with prosthetic material	53 (7.1)	10 (6.1)	43 (7.3)	0.730
Cyanotic CHD	7 (0.9)	3 (1.8)	4 (0.7)	0.177
CHD repaired with prosthetic material	29 (3.9)	3 (1.8)	26 (4.4)	0.169
CHD repaired with shunt or conduit	8 (1.1)	1 (0.6)	7 (1.2)	1.000
Heart transplant with valvulopathy	0 (0)	0 (0)	0 (0)	1.000
Moderate risk	223 (29.7)	46 (28.2)	177 (30.1)	0.699
Acquired valvular dysfunction	127 (16.7)	20 (12.3)	107 (18.2)	0.077
Congenital valve anomalies (incl. bicuspid aortic valve, mitral valve prolapse with insufficiency)	117 (15.6)	31 (19.0)	86 (14.6)	0.179
Rheumatic heart disease	1 (0.2)	0	1 (0.2)	1.000
HOCM	3 (0.4)	0 (0)	3 (0.5)	1.000
Ventricular septal defect	6 (0.8)	2 (1.2)	4 (0.7)	0.615
Patent ductus arteriosus	0 (0)	0 (0)	0 (0)	1.000
Low/unknown risk	60 (8.0)	3 (1.8)	57 (9.7)	<0.001
LVAD	5 (0.7)	0 (0)	5 (0.8)	0.591
Cardiac device (PM, ICD)	58 (7.7)	3 (1.8)	55 (9.3)	<0.001
No risk factor	184 (24.5)	49 (30.1)	135 (22.9)	0.082
Endocarditis episode				
Definite IE	625 (83.1)	135 (82.8)	490 (83.2)	0.906
Possible IE	127 (16.9)	28 (17.2)	99 (16.8)	0.906
Valve/device affected				
Aortic valve	330 (43.9)	71 (43.6)	259 (44.0)	1.000
Mitral valve	311 (41.4)	75 (46.0)	236 (40.1)	0.179
Aortic and mitral valve	69 (9.2)	14 (8.6)	55 (9.3)	0.879
Right-sided IE	93 (12.4)	23 (14.1)	70 (11.9)	0.424
Prosthetic valve	192 (25.5)	36 (22.1)	156 (26.5)	0.266
Cardiac device	97 (12.9)	10 (6.1)	87 (14.8)	0.003
Other	36 (4.8)	8 (4.9)	28 (4.8)	1.000
Causative pathogen				
*Staphylococcus aureus*	260 (34.6)	50 (30.7)	210 (35.7)	0.264
Coagulase-neg. Staphylococci	70 (9.3)	15 (9.2)	55 (9.3)	1.000
*Enterococcus* spp	77 (10.2)	16 (9.8)	61 (10.4)	1.000
*Streptococcus* spp	230 (30.6)	51 (31.3)	179 (30.4)	0.848
Oral *Streptococcus* spp	164 (21.8)	37 (22.7)	127 (21.6)	0.749
Other pathogens	113 (15.0)	29 (17.8)	84 (14.3)	0.267
Culture-negative	25 (3.3)	9 (5.5)	16 (2.7)	0.086
Clinical outcomes				
Surgery for IE	453 (60.2)	104 (63.8)	349 (59.3)	0.320
90-day mortality, % (95% CI)	16.1 (13.5-19.0)	8.6 (5.1-14.4)	18.1 (15.1-21.5)	
1-year mortality, % (95% CI)	22.3 (19.3-26.1)	15.0 (9.6-22.8)	24.5 (20.1-28.7)	0.013

Values are n (%) or median (IQR) unless otherwise indicated.

BMI = body mass index; CCI = Charlson comorbidity index; CHD = congenital heart disease; COPD = chronic obstructive pulmonary disease; HOCM = hypertrophic obstructive cardiomyopathy; ICD = implantable cardioverter defibrillator; IE = infective endocarditis; IQR = interquartile range; IVDU = intravenous drug use; LVAD = left ventricular assist device; ORL = otorhinolaryngology; PM = pacemaker.

### Proportion of streptococcal IE before and after the implementation of AP guidelines changes

In total, oral streptococci were identified as the causative pathogen in 164 cases (21.8%), and any streptococci in 230 cases (30.6%). The proportion of IE caused by oral or any streptococci remained comparable in the periods before and after the guideline update (oral streptococci: 22.7% vs 21.6%, *P* = 0.749; all streptococci: 31.3% vs 30.4%, *P* = 0.848). The Central Illustration depicts the proportion of IE attributed to oral streptococci across various risk groups. In patients with moderate-risk conditions, the percentage of IE episodes caused by oral streptococci increased from 24.4% to 39.0% following the guideline update (*P* = 0.083). Additionally, the proportion of episodes caused by any streptococci increased from 31.1% to 49.7% (*P* = 0.030). This stands in contrast to all other risk groups, where a decreasing trend was observed.

**Figure 2:**
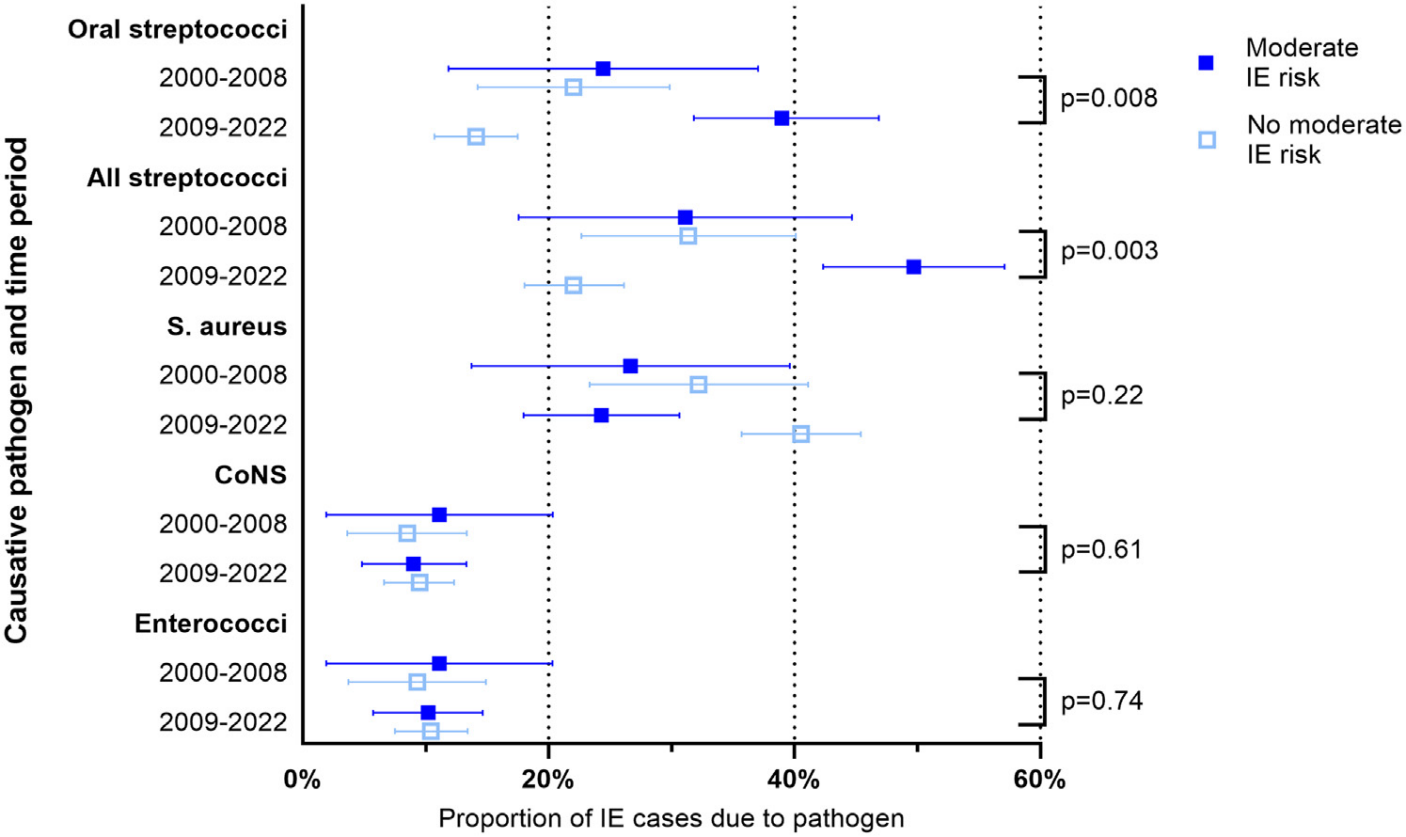
**Change in Proportion of IE Due to Different Pathogens Among the Moderate-Risk Group vs All Other Patients Before and After Change of AP Recommendations** The proportion of IE episodes due to different pathogens before and after the change in AP guidelines for moderate-risk patients versus all other patients as a baseline are displayed. Notably, the proportion of IE attributed to oral streptococci increased by 22.5% (95% CI: 5.9%-39.1%) in the moderate-risk group compared to the other patients following the guideline change. *P* values shown are for difference between means of change in proportion over time between moderate-risk and all other patients. AP = antibiotic prophylaxis; CoNS = coagulase-negative staphylococci; and other abbreviation as in Figure 1.

When examining differences between the two time periods for patients at moderate risk compared to all other risk categories (Figure 2), we observed among the moderate-risk group a 22.5% (95% CI: 5.9%-39.1%) and 27.9% (95% CI: 9.8%-46.0%) increase in the proportion of IE attributed to oral or any streptococci, respectively. Conversely, patients not at moderate risk experienced a notable rise in the proportion of IE attributed to *S. aureus*.

### Association of moderate-risk category and IE due to oral and any streptococci

**Figure 3:**
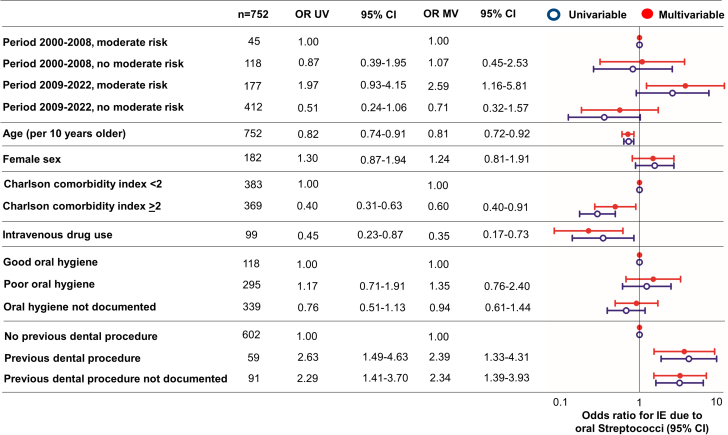
**Univariable and Multivariable Logistic Regression Model for OR of IE Due to Oral Streptococci** The results of univariable and multivariable logistic regression analyses assessing the risk of Infective Endocarditis (IE) caused by oral streptococci are presented. Following comprehensive adjustments for all variables listed, noteworthy positive associations were observed in individuals at moderate IE risk during period 2 compared to period 1, as well as those who underwent recent high-risk dental procedures. Conversely, negative associations were identified for intravenous drug users (IVDU) and individuals of higher age. MV = multivariable; UV = univariable.

Figure 3 illustrates both univariable and multivariable associations with IE attributed to oral streptococci. Univariable associations with IE due to oral streptococci included a documented high-risk dental procedure within 3 months prior to IE diagnosis (OR: 2.63 [95% CI: 1.49-4.63]), IVDU (OR: 0.45 [95% CI: 0.23-0.87]), CCI ≥2 (OR: 0.40 [95% CI: 0.31-0.63]), and age per 10 years older (OR: 0.82 [95% CI: 0.74-0.91]). After multivariable adjustment, patients at moderate risk exhibited a more than twofold increased risk of IE due to oral streptococci (OR: 2.59 [95% CI: 1.16-5.81]) in the period following the guideline change compared to before. Other associations that remained consistent after adjustment were high-risk dental procedure (OR: 2.39 [95% CI: 1.33-4.31]), IVDU (OR: 0.35 [95% CI: 0.17-0.73]), CCI ≥2 (OR: 0.60 [95% CI: 0.40-0.91]), and age (per 10 years older OR: 0.81 [95% CI: 0.72-0.92]).

Notable associations in the multivariable analysis were also identified with IE caused by any streptococci, such as being at moderate risk in period 2 compared to period 1 (OR: 2.66 [95% CI: 1.26-5.61]), recent dental procedure (OR: 1.85 [95% CI: 1.04-3.31]), and documented poor oral hygiene (OR: 1.75 [95% CI: 1.04-2.94]). IVDU, age, and higher CCI were negatively associated. All associations are shown in Supplemental Figure 1.

### Association of specific risk conditions with IE due to oral streptococci

In order to identify patients at higher risk of developing streptococcal IE, additional univariable logistic regression analyses were conducted for IE attributed to oral streptococci and any streptococci, respectively, during the whole time period. The following risk conditions were found to be associated with an increased risk of IE due to oral streptococci: cyanotic congenital heart disease (CHD) (OR: 9.21; 95% CI: 1.77-48.02) and congenital valve anomalies (OR: 2.52; 95% CI: 1.71-3.71). Conversely, the presence of a cardiac implantable electronic device and the absence of any identifiable risk factors were negatively associated (OR: 0.35; 95% CI: 0.19-0.62 and OR: 0.57; 95% CI: 0.36-0.89, respectively). For further details, please refer to Supplemental Figure 2. IE due to any streptococci was associated with cyanotic CHD (OR: 5.78; 95% CI: 1.11-30.0) and congenital valve anomalies (OR: 1.96; 95% CI: 1.36-2.84), while it was negatively associated with the presence of a prosthetic valve (OR: 0.58; 95% CI: 0.40-0.84) or a cardiac implantable electronic device (OR: 0.37; 95% CI: 0.23-0.59).

## Discussion

In our observational study involving 752 episodes of IE from 683 patients treated at a Swiss tertiary care center, we identified an association of IE caused by oral streptococci and the moderate-risk group of patients, particularly in the period following AP guideline change. This suggests a potential adverse effect of discontinuing AP in this population. Notably, recent invasive dental procedures independently correlated with oral streptococcal IE, highlighting a potential avenue for enhanced IE prevention with AP. Over the whole observation period, patients with cyanotic CHD or congenital valve anomalies were more predisposed to developing IE due to oral streptococci compared to those with other risk conditions.

Our study contributes to the extensive literature on this debated topic. In 2021, the AHA issued a statement examining the changes in incidence and mortality of oral streptococcal IE following the AP guideline change. The AHA reviewed 18 observational studies spanning from 2008 to 2019, presenting conflicting results—some found an overall increase in streptococcal IE incidence after the guideline change, while others did not.^19^ Many of these studies were health registry-based and did not consider the patients’ IE risk category, which we believe is crucial, given that the guideline change primarily affected patients at moderate risk. As demonstrated in our analysis, a specific rise in cases of oral streptococcal IE among moderate-risk patients following the cessation of AP may go unnoticed when evaluating the overall incidence in a population. In our study, there was no change in the overall proportion of oral streptococcal IE between the 2 periods, attributable to a declining incidence in other risk groups. Consequently, we argue that the observed increase in moderate-risk patients is not contradicted by previous studies.

Likewise, Thornhill et al investigated the effects of the AP guideline change across various risk groups in the United States.^30^ They identified an increase in IE incidence specifically among high- and moderate-risk patients, subsequent to a reduction in AP prescriptions within these risk categories. Despite a more pronounced decrease in AP prescriptions in the moderate-risk group, there was a smaller increase in IE incidence among moderate-risk patients compared to high-risk patients. Therefore, it was hypothesized that AP may be less effective in moderate-risk patients. Alternatively, they suggested that only a specific subgroup of individuals at moderate risk may derive benefits from AP. In our analysis, congenital valve anomaly was associated with oral streptococcal IE, highlighting this patient group as a promising target for AP. This is supported by a Spanish study by Zegri et al, which found that patients with a BAV or MVP had a significantly higher risk of IE attributed to oral streptococci of odontological origin compared to other risk groups.^31^ In light of recent findings that patients with congenital valve anomalies, currently classified as moderate risk, face a similar risk of developing and succumbing to IE as those in several high-risk categories,^20^ we advocate for a careful reassessment of the indication for AP in this specific patient group.

To the best of our knowledge, this study is the first to assess the impact of the 2008 AP guideline change by concentrating on the risk of oral streptococcal IE within the affected moderate-risk group. We utilized physician-adjudicated data obtained through direct access to hospital health records, enabling a more thorough collection of information on IE risk, microbiology, and recent dental procedures than in previous registry-based studies. Moreover, we have thoroughly gathered information on AP administration before hospital admission.

### Study Limitations

First, similar to numerous other studies, we were unable to compare disease prevalence among demographically similar patients who underwent a dental procedure but did not develop IE. Conducting a traditional individual-based randomized controlled trial to demonstrate the efficacy of AP for IE prevention is deemed unethical. This is primarily attributed to the low incidence of IE, the diverse range of predisposing heart conditions, and the complexity of dental procedures. Meanwhile, the ongoing French trial PROPHETS (NCT05613933) is hypothesizing that dentists within specific health territories, assigned to the intervention group, will increase AP prescriptions for patients with prosthetic valves and a history of prior IE. The objective is to reduce the incidence of oral streptococcal IE compared to the control group, thereby substantiating the efficacy of AP in preventing IE. Until the release of the PROPHETS trial, we will still have to rely on observational studies concentrating on IE in patients with moderate risk conditions. Second, although we have demonstrated an increase of streptococcal IE cases within the moderate-risk group, it is crucial to acknowledge that other factors, not solely the modification of the AP guidelines, could potentially contribute to this increase. Recent nationwide studies in Sweden and England, utilizing extensive databases, have revealed a connection between nondental invasive procedures and the risk of IE. The Swedish case-crossover study, analyzing 7,013 IE patients, identified increased IE risks associated with various procedures, including coronary artery bypass grafting, skin procedures, wound management, transfusions, dialysis, bone marrow puncture, and certain endoscopies, notably bronchoscopy.^32^ Similarly, the English study, examining nearly 15,000 IE hospital admissions,^33^ found an association between IE and nondental invasive procedures such as permanent pacemaker and defibrillator implantation, upper and lower gastrointestinal endoscopy, bone marrow biopsy, bronchoscopy, and blood transfusions/red cell/plasma exchange. A recent systematic review aggregated trends in IE incidence from various nationwide population-based studies in Europe. The study revealed a concerning 4% annual increase in IE incidence, leading to a doubling between 2000 and 2018.^9^ The rise is likely attributed to factors such as improved diagnosis, changes in epidemiology and risk factors, restrictions in AP use per updated guidelines, and enhancements in coding practices in the 21st century.^9^ Similarly, our study spans from 2000 to 2022, during which advanced imaging and microbiological techniques evolved, potentially altering diagnostic practices and accuracy of the different versions of the Duke criteria.^23^^,^^24^ Moreover, due to the retrospective/prospective design of our study, there is a difference in the number of streptococcal IE cases between the two periods, which could potentially introduce bias. As there might be a documentation gap regarding dental procedures and AP administration in the patients, we cannot completely rule out the possibility that participants sought care at other institutions, potentially leading to underreporting by participants or care providers. Therefore, formal record linkage with other hospitals is essential for future studies. Given the observational nature of the study, we cannot eliminate the chance of residual confounding, highlighting the importance of multicentered studies to validate the findings of this research. The retrospective data collection also necessitated our reliance on the dental assessment information provided in the medical records. Lastly, our study was conducted in Switzerland, which may limit the generalizability of our results since relevant factors such as dental care models and IVDU rates may differ from other countries.

## Conclusions

We identified an association between oral streptococcal IE and the moderate-risk group, particularly in the period following the AP guideline change. Hence, we advocate for a thoughtful reassessment of AP indications in moderate-risk patients, especially those with congenital valve anomalies such as BAV or MVP with regurgitation. Furthermore, regular annual dental check-up for prophylaxis and comprehensive knowledge transfer regarding IE for patients at moderate risk are fundamental. Moreover, in light of the new data on the association between NDPI and IE, further studies on the use of AP for IE prevention are essential. However, the broader implications of AP should be considered. AP can alter oral flora, promote antibiotic resistance, and decrease antimicrobial sensitivity over time. Additionally, antibiotics can cause adverse effects, and their costs must be weighed against benefits.

PERSPECTIVES **COMPETENCY IN MEDICAL KNOWLEDGE:** Our findings indicate that abstaining from AP prior to invasive dental procedures in moderate-risk patients, particularly those with a BAV or MVP, could result in a rise in cases of oral streptococcal IE.

**TRANSLATIONAL OUTLOOK:** Our study underscores the imperative for additional research to investigate the repercussions of discontinuing AP in moderate-risk patients. Furthermore, there is a pressing need to reassess the appropriateness of AP indications within specific populations at risk.

## Funding support and author disclosures

This study was funded within the framework of the 10.13039/501100001711Swiss National Science Foundation grants 320030_184918/1 and 32003B_218351/1 (to Dr Hasse). Additional support was received from the Clinical Research Priority Program of the University of Zurich for the CRPP Precision medicine for bacterial infections (to Dr Hasse, Dr Zinkernagel) and the Nakao Foundation Grant for Worldwide Oral Health (to Dr Özcan, Dr Carrel). The authors have reported that they have no relationships relevant to the contents of this paper to disclose.
