# Supplementary material for: Increase in Oral Streptococcal Endocarditis Among Moderate-Risk Patients: Impact of Guideline Changes on Endocarditis Prevention
Source: JACC Adv. 2024 Sep 6;3(10):101266. doi: 10.1016/j.jacadv.2024.101266 (PMC11406034; doi:10.1016/j.jacadv.2024.101266)

Supplementary material

Supplementary Figure 1: Uni- and multivariable logistic regression model for odds (OR) of IE due to any streptococci

The results of both univariable and multivariable analyses examining the risk of infective endocarditis (IE) caused by any streptococci are presented. Notably, significant positive associations, following multivariable adjustment, were identified for individuals classified at moderate IE risk in period 2 compared to period 1, recent high-risk dental procedures, and documented poor dental hygiene.

Abbreviations: IE, infective endocarditis; OR, odds ratio; UV, univariable; MV, multivariable

	n=752	OR UV	95% CI	OR MV	95% CI
**Period 2000-2008, moderate risk**	45	1.00		1.00	
**Period 2000-2008, no moderate risk**	118	1.01	0.48-2.14	1.27	0.58-2.80
**Period 2009-2022, moderate risk**	177	2.19	1.09-4.40	2.66	1.26-5.61
**Period 2009-2022, no moderate risk**	412	0.63	0.32-1.23	0.82	0.40-1.70
**Age (per 10 years older)**	752	0.88	0.81-0.98	0.88	0.79-0.98
**Female sex**	182	1.41	0.98-2.03	1.40	0.96-2.01
**Charlson comorbidity index <2**	383	1.00		1.00	
**Charlson comorbidity index ≥2**	369	0.52	0.38-0.71	0.62	0.43-0.86
**Intravenous drug use**	99	0.43	0.23-0.78	0.32	0.17-0.61
**Good oral hygiene**	118	1.00		1.00	
**Poor oral hygiene**	295	1.35	0.86-2.14	1.74	1.04-2.94
**Oral hygiene not documented**	339	0.90	0.63-1.28	1.17	0.80-1.71
**No previous dental procedure**	602	1.00		1.00	
**Previous dental procedure**	59	2.02	1.17-3.49	1.85	1.04-3.31
**Previous dental procedure not documented**	91	1.60	1.00-2.54	1.49	0.92-2.42

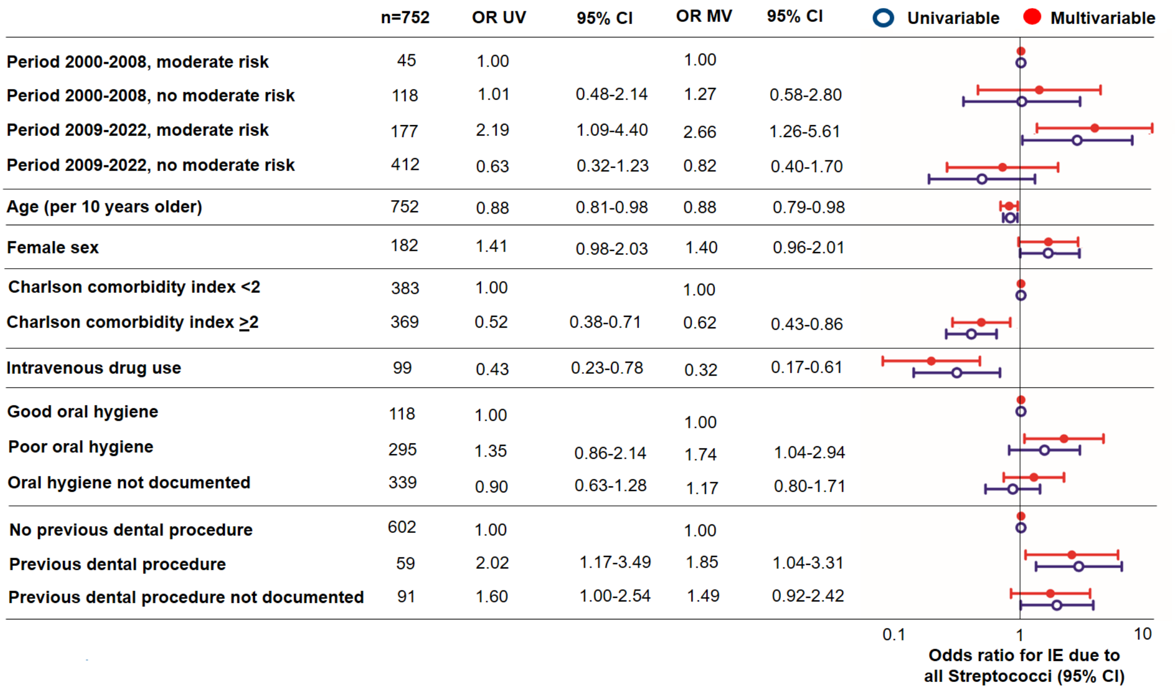

Supplementary Figure 2: Univariable logistic regression models for odds ratios (ORs) of IE due to oral streptococci for various underlying risk conditions

The risk of oral streptococcal infective endocarditis (IE) varies depending on the underlying predisposing IE risk condition. Cyanotic congenital heart conditions and congenital valve anomalies are linked to a heightened risk of streptococcal IE. Conversely, the risk is diminished in patients with a cardiac device or those lacking any identifiable IE risk factors. Abbreviations: CHD, congenital heart disease; HOCM, hypertrophic obstructive cardiomyopathy; IE, infective endocarditis; LVAD, left ventricular assist device; PDA, persistent ductus arteriosus; VSD, ventricular septal defect

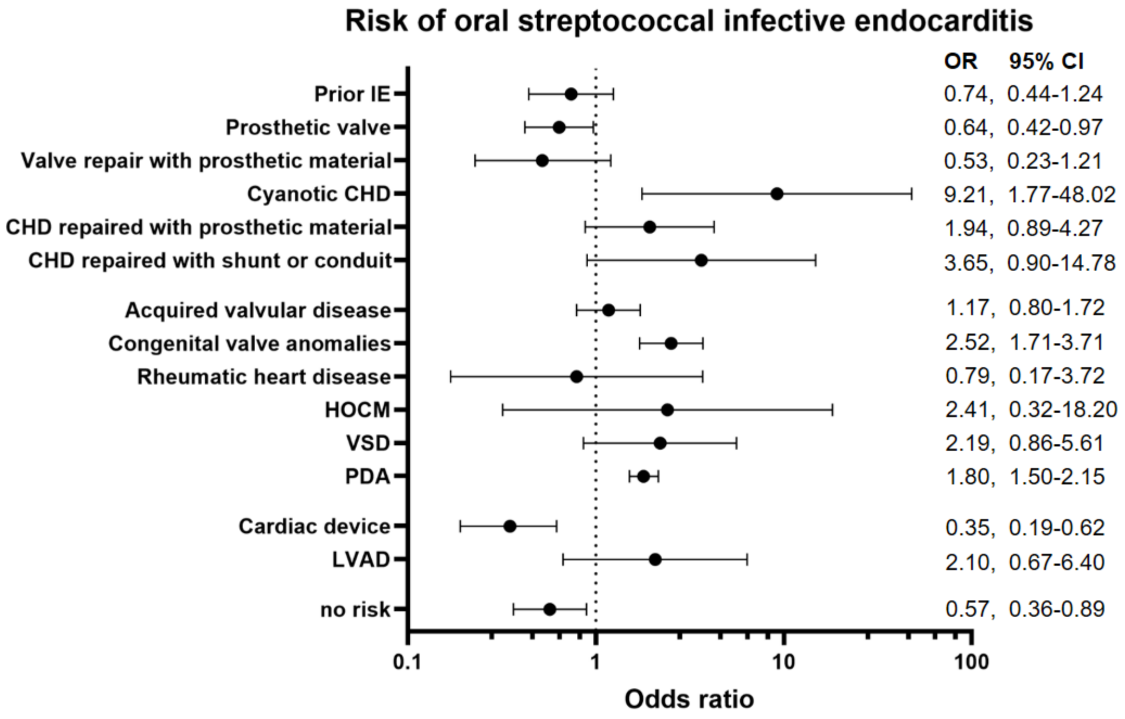

Supplement: Supplementary material [file mmc1.pdf]
